# Vitamin D supplementation may be beneficial in improving the prognosis of patients with sepsis-associated acute kidney injury in the intensive care unit: a retrospective study

**DOI:** 10.3389/fmed.2024.1453522

**Published:** 2024-12-02

**Authors:** Jie Sun, Yan Wang, Jue Wang, Hongwei Wu, Zhefeng Xu, Dongsheng Niu

**Affiliations:** ^1^Department of Emergency Medicine, Jincheng People's Hospital, Jincheng, China; ^2^Department of Respiratory and Critical Care Medicine, Jincheng General Hospital, Jincheng, China

**Keywords:** vitamin D supplementation, mortality, SA-AKI, MIMIC-IV, intensive care unit

## Abstract

**Background:**

Vitamin D, an essential fat-soluble micronutrient, exerts diverse physiological effects including the regulation of calcium ion homeostasis, modulation of immune response, and enhancement of resistance against infectious pathogens. Empirical investigations have elucidated an association between inadequate levels of vitamin D and adverse clinical outcomes in critically ill cohorts, with a noteworthy prevalence of vitamin D deficiency observed among patients afflicted with acute kidney injury (AKI). In the context of this retrospective inquiry, our aim was to assess the potential correlation between vitamin D supplementation administered during admission to the intensive care unit (ICU) and the improvement of outcomes specifically in cases of severe AKI.

**Methods:**

This study utilized data from the Medical Information Mart for Intensive Care IV (MIMIC-IV), a repository of ICU patient records from Beth Israel Deaconess Medical Center (BIDMC) in the United States. We focused on patients diagnosed with epsis-associated acute kidney injury (SA-AKI), dividing them into those who received vitamin D supplementation during their ICU admission and those who did not. Our primary analysis evaluated in-hospital mortality using various statistical methods, such as Kaplan–Meier survival curves, Cox proportional hazards regression models, and subgroup analyses. To enhance the robustness of our findings, we used propensity score matching (PSM) to reduce potential biases. Secondary outcomes included 28-day, 90-day mortality rates and norepinephrine-free days at 28 days.

**Results:**

In this investigation, a cohort of 11,896 individuals diagnosed with SA-AKI was studied. Among them, 2,724 patients received vitamin D supplementation (the vitamin D group) while 9,172 did not (the no-vitamin D group). Kaplan–Meier survival analysis indicated a significant difference in survival probabilities between the two cohorts. Upon adjusting for potential confounders using Cox regression modeling, a notably decreased risk of hospitalization and ICU mortality was observed in the vitamin D group compared to the no-vitamin D group, with an adjusted risk ratio for in-hospital mortality of 0.56 (95% CI: 0.5–0.63). These findings were consistent following PSM and subsequent adjustments for propensity score, pairwise algorithm (PA), and overlapping weights (OW) analyses, yielding hazard ratios ranging from 0.53 to 0.59, all with *p*-values <0.001. Notably, E-value analyses underscored the robustness of these results against potential unmeasured confounders.

**Conclusion:**

This study suggests that vitamin D supplementation may be associated with a reduced in-hospital mortality rate among SA-AKI patients in the ICU. Furthermore, the 28-day, 90-day mortality rates and norepinephrine days were significantly reduced in the group receiving vitamin D supplementation.

## 1 Introduction

Sepsis is defined as a state of organ dysfunction resulting from the dysregulation of the host’s immune response to infection, thus posing a considerable risk of morbidity and mortality in critically ill patients (1). Notably, the kidney assumes a primary and early role in sepsis, with acute kidney injury (AKI) being a comprehensive clinical syndrome characterized by a sudden decline in renal function, encompassing various manifestations beyond the scope of acute renal failure alone (2). Importantly, the occurrence of sepsis-associated acute kidney injury (SA-AKI) significantly escalates the likelihood of in-hospital mortality, the development of chronic kidney disease, and the need for renal replacement therapy (3, 4).

Studies have elucidated the pleiotropic effects of vitamin D on immune function, endothelial and mucosal integrity, and glucose metabolism, in addition to its established role in calcium homeostasis regulation (5). Furthermore, numerous investigations have underscored the associations between vitamin D deficiency and heightened mortality and morbidity rates across diverse chronic conditions, encompassing coronary artery disease, tuberculosis, malignant neoplasms, and chronic kidney disease (6).

In the context of critically ill patients, vitamin D insufficiency has been correlated with a significantly increased incidence of sepsis and organ dysfunction, both of which are implicated in elevated mortality rates (7, 8). However, despite an extensive corpus of literature interrogating the nexus between vitamin D and sepsis in critically ill cohorts, scant attention has been devoted to investigating SA-AKI. Remarkably, the biologically active form of vitamin D is synthesized within the renal proximal tubule mitochondria, where 1α-hydroxylase catalyzes the conversion of 25-OH vitamin D to its biologically active metabolite, 1,25-dihydroxy vitamin D (9). The principal mechanism underlying SA-AKI involves renal ischemia–reperfusion injury. Interestingly, evidence suggests that the severity of this injury correlates with deficiencies in vitamin D receptor expression and the downregulation of P21 (10). The primary aim of this study was to assess the potential efficacy of vitamin D supplementation in improving clinical outcomes among intensive care unit (ICU) SA-AKI patients.

## 2 Methods

### 2.1 Data sources and setting

A population-based cohort study utilized the Critical Care Database from the Medical Information Mart for Intensive Care (MIMIC-IV, version 2.2), an extension of MIMIC-III. This database encompassed 76,540 ICU admissions spanning 2008 to 2019. Approval to access the database (certification number 54835759) was obtained by Jie Sun. Data were de-identified prior to use, and both the institutional review boards of Massachusetts Institute of Technology (No. 0403000206) and Beth Israel Deaconess Medical Center (2001-P-001699/14) approved its utilization for research. Strict adherence to ethical regulations governing research data use was maintained throughout the study.

### 2.2 Study population

**Figure 1:**
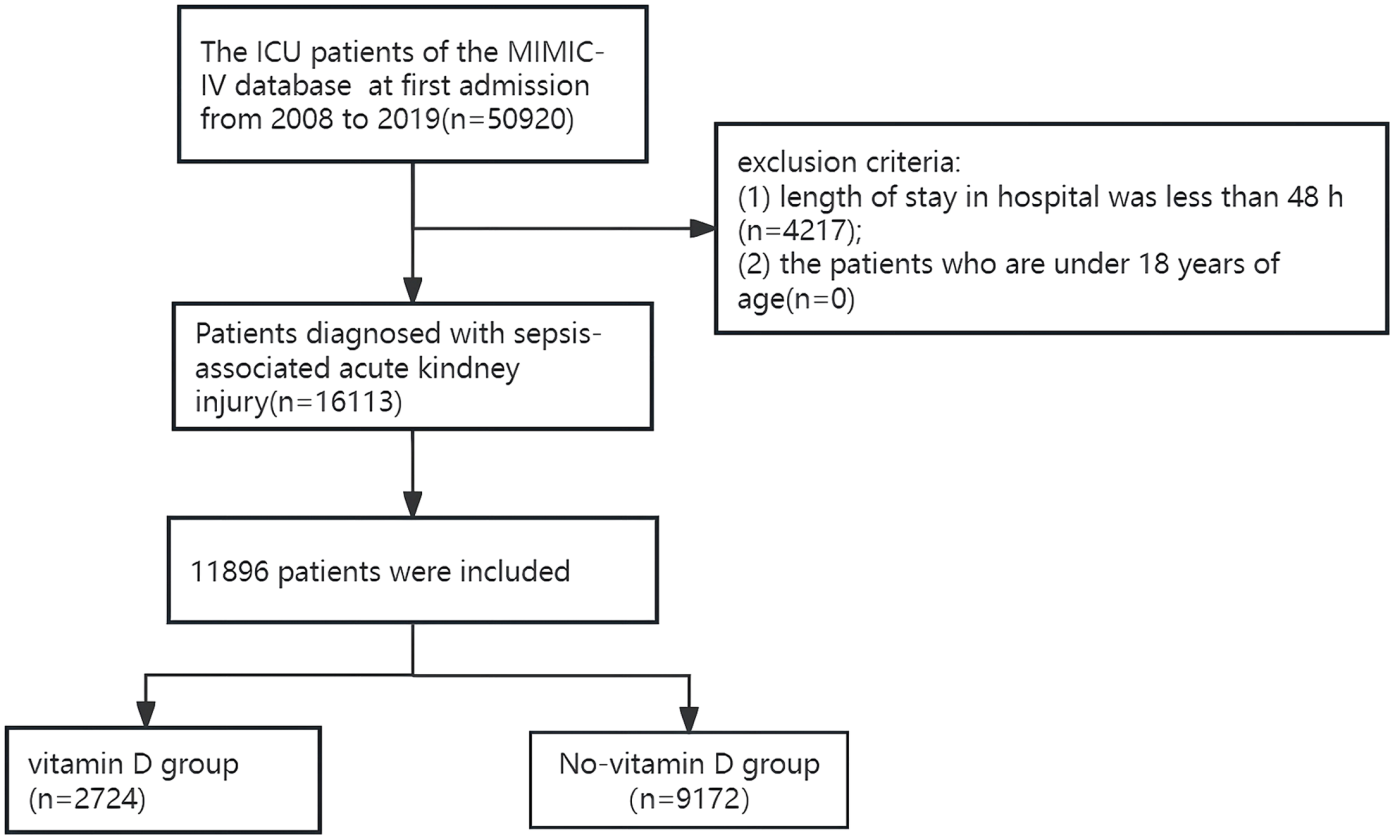
Flowchart of participant selection.

A total of 50,920 patients who were admitted to the ICU for the first time were identified from the MIMIC database. The flow chart and number of patients in this study are shown in Figure 1. Only patients diagnosed with SA-AKI were included. The study excluded individuals with a hospitalization duration of less than 48 h and those under 18 years of age. The study subjects conformed to the sepsis-3 criteria from the Third International Consensus for diagnosing sepsis and septic shock (11). AKI identification and classification followed the 2012 Kidney Disease: Improving Global Outcomes (KDIGO) guidelines, AKI was defined as a serum creatinine (sCr) increase of ≥0.3 mg/dL (26.5 μmol/L) within 48 h, sCr elevation to ≥1.5 times baseline in the past 7 days, or urine output <0.5 mL/kg/h over a 6-h period (12). If the baseline sCr level was not documented before ICU admission, the first recorded sCr level post-admission was used as the baseline reference.

### 2.3 Main exposure

The primary independent variable under consideration was the administration of vitamin D supplementation (via both intravenous and oral routes) after admission to the ICU, which led to categorizing patients into a vitamin D cohort and a non-vitamin D cohort.

### 2.4 Covariates

In this study, we measured patient characteristics that have previously been demonstrated to influence changes in mortality rates of SA-AKI. The variables examined in this study included demographic factors such as age, gender, and race, alongside vital signs and a comprehensive range of laboratory tests. Vital signs were recorded as average values within the first 24 h post-ICU admission, while laboratory indicators were derived from the worst values observed during the same period. The laboratory tests encompassed parameters including hemoglobin, white blood cell (WBC) count, platelet count, bicarbonate, creatinine, sodium, potassium, calcium, chloride, blood urea nitrogen (BUN), prothrombin time (PT), activated partial thromboplastin time (APTT), serum lactate, and glucose. Additionally, comorbidities including myocardial infarction, congestive heart failure, rheumatic disease, renal disease, chronic pulmonary disease, peptic ulcer disease, mild liver disease, severe liver disease, malignant cancer, hypertension, and diabetes mellitus were recorded, alongside the charlson comorbidity index. AKI staging was determined in accordance with the KDIGO criteria. Throughout the treatment process, the use of invasive mechanical ventilation, vasoactive drugs, renal replacement therapy (RRT) and disease severity scores, such as the Sequential Organ Failure Assessment (SOFA) Score and the Simplified Acute Physiology Score (SAPS) II, were documented, we used the SOFA score and SAPS II score from the first day after admission to the ICU as assessment indicators.

### 2.5 Primary outcome and secondary outcomes

The primary outcome of the study was in-hospital mortality, Secondary outcomes include 28-day mortality, 90-day mortality and norepinephrine-free days within 28 days after ICU admission.

### 2.6 Statistical analysis

Descriptive analyses were performed for all participants. Categorical variables were presented as frequencies and percentages, while continuous variables were expressed as means with standard deviations (SD) for normally distributed data, or medians with interquartile ranges (IQR) for skewed data. Statistical tests included the chi-square test for categorical variables, the *t*-test for normally distributed continuous variables, and the Kruskal-Wallis test for non-normally distributed continuous variables. Kaplan–Meier estimates and log-rank tests were employed to analyze survival curves.

To minimize potential bias resulting from confounding factors among various groups, we calculated propensity scores using logistic regression and applied a 1:1 nearest neighbor matching algorithm with a caliper width of 0.01. The variables selected for this analysis included age, sex, race, vital signs, laboratory test results, comorbidities, and levels of disease verification, as informed by the existing literature. The standardized mean difference (SMD) was utilized to assess the effectiveness of the propensity score matching, with a threshold of less than 0.1 considered acceptable.

In the propensity score matched (PSM) cohort, we conducted a two-sided *t*-test to evaluate differences in the secondary outcome between the two groups. To investigate the relationship between vitamin D supplementation and in-hospital mortality, we performed both univariate and multivariate Cox regression analyses, with the multivariate analysis incorporating all variables used in generating the propensity scores. The estimated propensity scores were utilized as weights to adjust for intergroup differences. A weighted cohort was created using the pairwise algorithm (PA) (13) and overlapping weights (OW) (14) models.

All analyses were carried out using the statistical software package R version 3.3.2^1^ and the free statistical software version 1.4 of R (15). Two-tailed tests were performed on www.R-project.org, with *p*-values less than 0.05 regarded as statistically significant.

### 2.7 Sensitivity analysis and subgroup analysis

We performed a sensitivity analysis on patients who developed AKI within the 48-h period preceding ICU admission. Furthermore, we conducted subgroup analyses categorized by age, sex, SOFA score, SAPS II score, use of vasoactive medications, presence of invasive ventilation, as well as comorbid conditions including hypertension, diabetes mellitus, renal disease, chronic pulmonary disease, and congestive heart failure. The possibility of unmeasured confounders between the vitamin D supplementation group and in-hospital mortality was assessed by calculating E-values (16).

## 3 Results

### 3.1 Participants

A total of 50,920 patients who were admitted to the ICU for the first time were identified from the MIMIC database. The flow chart and number of patients in this study are shown in Figure 1. Only patients diagnosed with SA-AKI were included. Excluded from the study were patients who had a hospital stay in the ICU for less than 48 h, and patients who were under 18 years old. A total of 11,896 patients with SA-AKI were included in the study, among which 2,724 patients received vitamin D supplementation, and 9,172 patients did not receive vitamin D supplementation.

### 3.2 Baseline characteristics

The baseline characteristics of all subjects are presented in Table 1. The mean age of the participants was 67.1 ± 16.0 years, with 57.7% (6,862 individuals) being male. BUN levels were elevated in the vitamin D supplementation group. Additionally, there was a higher prevalence and increased Charlson comorbidity index for conditions such as congestive heart failure, chronic pulmonary disease, rheumatic disease, severe liver disease, diabetes mellitus, hypertension, renal disease comorbidities, and malignant cancer in this group. Furthermore, the odds of requiring RRT during admission were greater in the vitamin D supplementation group, whereas the need for mechanical ventilation during admission was more prevalent in the group that did not receive vitamin D supplementation. The baseline characteristics of the two groups after PSM are balanced in Table 1.

**Table 1:** Baseline characteristics of participants.

Covariate	Unmatched patients			SMD	*p* value	Propensity-score–matched patients			SMD	*p* value
	Total	No vitamin D	Vitamin D			Total	No vitamin D	Vitamin D		
*n*	11,896	9,172	2,724			5,392	2,696	2,696		
Age (years)	67.1 ± 16.0	66.3 ± 16.2	69.9 ± 14.7	0.234	< 0.001	69.9 ± 14.7	69.9 ± 14.8	70.0 ± 14.7	0.003	0.901
Male, sex, *n* (%)	6,862 (57.7)	5,523 (60.2)	1,339 (49.2)	0.224	< 0.001	2,654 (49.2)	1,319 (48.9)	1,335 (49.5)	0.012	0.663
Race, *n* (%)				0.361	< 0.001				0.026	0.921
White	7,851 (66.0)	5,890 (64.2)	1961 (72)			3,916 (72.6)	1972 (73.1)	1944 (72.1)		
Black	955 (8.0)	636 (6.9)	319 (11.7)			606 (11.2)	298 (11.1)	308 (11.4)		
Asia	284 (2.4)	210 (2.3)	74 (2.7)			142 (2.6)	68 (2.5)	74 (2.7)		
Hispanic	261 (2.2)	204 (2.2)	57 (2.1)			109 (2.0)	52 (1.9)	57 (2.1)		
Other	2,545 (21.4)	2,232 (24.3)	313 (11.5)			619 (11.5)	306 (11.4)	313 (11.6)		
Vital Signs, mean (SD)										
Heart rate (bpm)	87.5 ± 16.6	87.7 ± 16.6	86.9 ± 16.8	0.051	0.019	87.0 ± 16.5	87.2 ± 16.1	86.9 ± 16.8	0.019	0.496
MAP (mmHg)	76.6 ± 10.2	77.0 ± 10.3	75.5 ± 9.9	0.141	< 0.001	75.4 ± 9.9	75.2 ± 10.0	75.5 ± 9.8	0.034	0.218
Respiratory rate (bpm)	19.9 ± 4.1	19.9 ± 4.1	19.9 ± 4.1	0.02	0.35	19.9 ± 4.1	19.9 ± 4.1	19.9 ± 4.1	0.003	0.908
Temperature (°C)	36.9 ± 0.7	36.9 ± 0.7	36.8 ± 0.6	0.085	< 0.001	36.8 ± 0.6	36.8 ± 0.6	36.9 ± 0.6	0.01	0.709
SPO2 (%)	97.1 ± 2.2	97.1 ± 2.2	96.9 ± 2.2	0.081	< 0.001	96.9 ± 2.2	96.9 ± 2.2	96.9 ± 2.2	0.004	0.878
Laboratory tests										
Hemoglobin, g/dL	9.9 ± 2.2	10.0 ± 2.2	9.4 ± 2.1	0.264	< 0.001	9.4 ± 2.1	9.4 ± 2.1	9.4 ± 2.1	0.006	0.828
Platelets, 10^9^/L	160.0 (109.0, 225.0)	161.0 (111.0, 224.0)	158.5 (103.0, 228.0)	0.015	0.094	158.0 (104.0, 227.0)	156.0 (105.0, 226.0)	159.0 (103.0, 228.0)	0.006	0.834
White Blood Cell,10^9^/L	14.4 (10.4, 19.4)	14.6 (10.6, 19.6)	13.8 (9.7, 18.9)	0.03	< 0.001	13.8 (9.9, 19.0)	13.9 (10.2, 19.0)	13.8 (9.7, 18.9)	0.005	0.225
Bicarbonate, mmol/L	24.1 ± 4.5	24.1 ± 4.4	24.2 ± 4.8	0.005	0.8	24.2 ± 4.7	24.2 ± 4.6	24.2 ± 4.8	0.002	0.929
BUN, mg/dL	24.0 (17.0, 40.0)	23.0 (16.0, 38.0)	29.0 (19.0, 47.0)	0.239	< 0.001	28.0 (18.0, 47.0)	28.0 (18.0, 48.0)	29.0 (18.0, 47.0)	0.01	0.723
Calcium, mmol/L	8.5 ± 0.9	8.5 ± 0.9	8.6 ± 1.0	0.113	< 0.001	8.6 ± 1.0	8.6 ± 0.9	8.6 ± 1.0	0.007	0.79
Chloride, mmol/L	106.4 ± 6.8	106.7 ± 6.7	105.6 ± 7.0	0.164	< 0.001	105.6 ± 7.0	105.6 ± 7.0	105.6 ± 7.0	0.007	0.795
Sodium, mmol/L	140.2 ± 5.4	140.3 ± 5.4	139.7 ± 5.5	0.101	< 0.001	139.7 ± 5.5	139.7 ± 5.5	139.8 ± 5.5	0.01	0.718
Potassium, mmol/L	4.7 ± 0.9	4.7 ± 0.9	4.7 ± 0.9	0.056	0.009	4.7 ± 0.9	4.8 ± 0.9	4.7 ± 0.9	0.018	0.504
Serum creatinine, mg/dL	1.2 (0.9, 2.0)	1.2 (0.9, 1.9)	1.3 (0.9, 2.3)	0.175	< 0.001	1.3 (0.9, 2.3)	1.3 (0.9, 2.4)	1.3 (0.9, 2.3)	0.02	0.195
Glucose, mmol/L	152.0 (122.0, 203.0)	151.0 (122.0, 201.0)	154.0 (122.0, 211.0)	0.057	0.008	154.0 (122.0, 211.0)	154.0 (122.0, 211.0)	154.0 (123.0, 211.0)	0.006	0.848
PT, s	15.1 ± 6.1	14.9 ± 5.7	15.9 ± 7.3	0.156	< 0.001	15.9 ± 7.0	15.9 ± 7.1	15.8 ± 6.9	0.013	0.763
APTT, s	31.8 ± 11.1	31.5 ± 10.7	33.0 ± 12.4	0.13	< 0.001	33.0 ± 12.7	33.1 ± 13.1	32.8 ± 12.2	0.02	0.764
Serum lactate, mmol/L	2.3 (1.5, 3.6)	2.3 (1.5, 3.6)	2.2 (1.4, 3.7)	0.027	0.027	2.2 (1.4, 3.6)	2.2 (1.5, 3.5)	2.2 (1.4, 3.6)	<0.001	0.559
Comorbidities, *n* (%)										
Myocardial infarction, *n* (%)	2,299 (19.3)	1775 (19.4)	524 (19.2)	0.003	0.893	1,060 (19.7)	537 (19.9)	523 (19.4)	0.013	0.631
Congestive heart failure, *n* (%)	4,019 (33.8)	2,929 (31.9)	1,090 (40)	0.169	< 0.001	2,141 (39.7)	1,064 (39.5)	1,077 (39.9)	0.01	0.717
Chronic pulmonary disease, *n* (%)	3,326 (28.0)	2,492 (27.2)	834 (30.6)	0.076	< 0.001	1,652 (30.6)	830 (30.8)	822 (30.5)	0.006	0.813
Rheumatic disease, *n* (%)	432 (3.6)	255 (2.8)	177 (6.5)	0.177	< 0.001	325 (6.0)	162 (6)	163 (6)	0.002	0.954
Peptic ulcer disease, *n* (%)	366 (3.1)	282 (3.1)	84 (3.1)	0.001	0.981	173 (3.2)	90 (3.3)	83 (3.1)	0.015	0.589
Mild liver disease, *n* (%)	1885 (15.8)	1,372 (15)	513 (18.8)	0.104	< 0.001	995 (18.5)	495 (18.4)	500 (18.5)	0.005	0.861
Severe liver disease, *n* (%)	940 (7.9)	630 (6.9)	310 (11.4)	0.157	< 0.001	597 (11.1)	300 (11.1)	297 (11)	0.004	0.896
Diabetes, *n* (%)	3,753 (31.5)	2,717 (29.6)	1,036 (38)	0.178	< 0.001	2070 (38.4)	1,037 (38.5)	1,033 (38.3)	0.003	0.911
Hypertension, *n* (%)	6,489 (54.5)	4,842 (52.8)	1,647 (60.5)	0.155	< 0.001	3,233 (60.0)	1,607 (59.6)	1,626 (60.3)	0.014	0.597
Renal disease, *n* (%)	2,792 (23.5)	1904 (20.8)	888 (32.6)	0.27	< 0.001	1739 (32.3)	871 (32.3)	868 (32.2)	0.002	0.93
Malignant cancer, *n* (%)	1,552 (13.0)	1,168 (12.7)	384 (14.1)	0.04	0.064	751 (13.9)	369 (13.7)	382 (14.2)	0.014	0.609
Charlson comorbidity index	6.1 ± 2.9	5.9 ± 2.9	6.7 ± 2.7	0.304	< 0.001	6.7 ± 2.7	6.7 ± 2.7	6.7 ± 2.8	0.02	0.537
Scoring system										
SAPS II	42.5 ± 14.2	42.2 ± 14.3	43.7 ± 13.7	0.108	< 0.001	43.6 ± 13.7	43.6 ± 13.8	43.6 ± 13.7	0.002	0.941
SOFA	3.0 (2.0, 5.0)	3.0 (2.0, 5.0)	3.0 (2.0, 5.0)	0.071	< 0.001	3.0 (2.0, 5.0)	3.0 (2.0, 5.0)	3.0 (2.0, 5.0)	0.005	0.857
AKI stage, *n* (%)				0.115	< 0.001				0.025	0.862
1	2,269 (19.1)	1811 (19.7)	458 (16.8)			904 (16.8)	447 (16.6)	457 (17)		
2	5,658 (47.6)	4,408 (48.1)	1,250 (45.9)			2,459 (45.6)	1,218 (45.2)	1,241 (46)		
3	3,969 (33.4)	2,953 (32.2)	1,016 (37.3)			2029 (37.6)	1,031 (38.2)	998 (37)		
GCS	10.7 ± 4.2	10.5 ± 4.2	11.2 ± 3.9	0.155	0.001	11.2 ± 3.9	11.1 ± 4.0	11.2 ± 3.9	0.007	0.798
RRT	1,550 (13.0)	1,105 (12)	445 (16.3)	0.123	0.001	873 (16.2)	446 (16.5)	427 (15.8)	0.019	0.482
Vasopressors use, *n* (%)	1,400 (11.8)	1,052 (11.5)	348 (12.8)	0.04	0.063	694 (12.9)	353 (13.1)	341 (12.6)	0.013	0.626
Invasive ventilation, *n* (%)	8,216 (69.1)	6,588 (71.8)	1,628 (59.8)	0.256	0.001	3,239 (60.1)	1,628 (60.4)	1,611 (59.8)	0.013	0.636

MAP, Mean Arterial Pressure; SPO2, pulse oxygen saturation; AKI, acute kidney injury; BUN, blood urea nitrogen; SOFA, sequential organ failure assessment; SAPS II, simplified acute physiology score II; RRT, Renal Replacement Therapy; GCS, Glasgow Coma Scale; PT, Prothrombin Time; APTT, Activated Partial Thromboplastin Time.

### 3.3 Primary outcome

**Figure 2:**
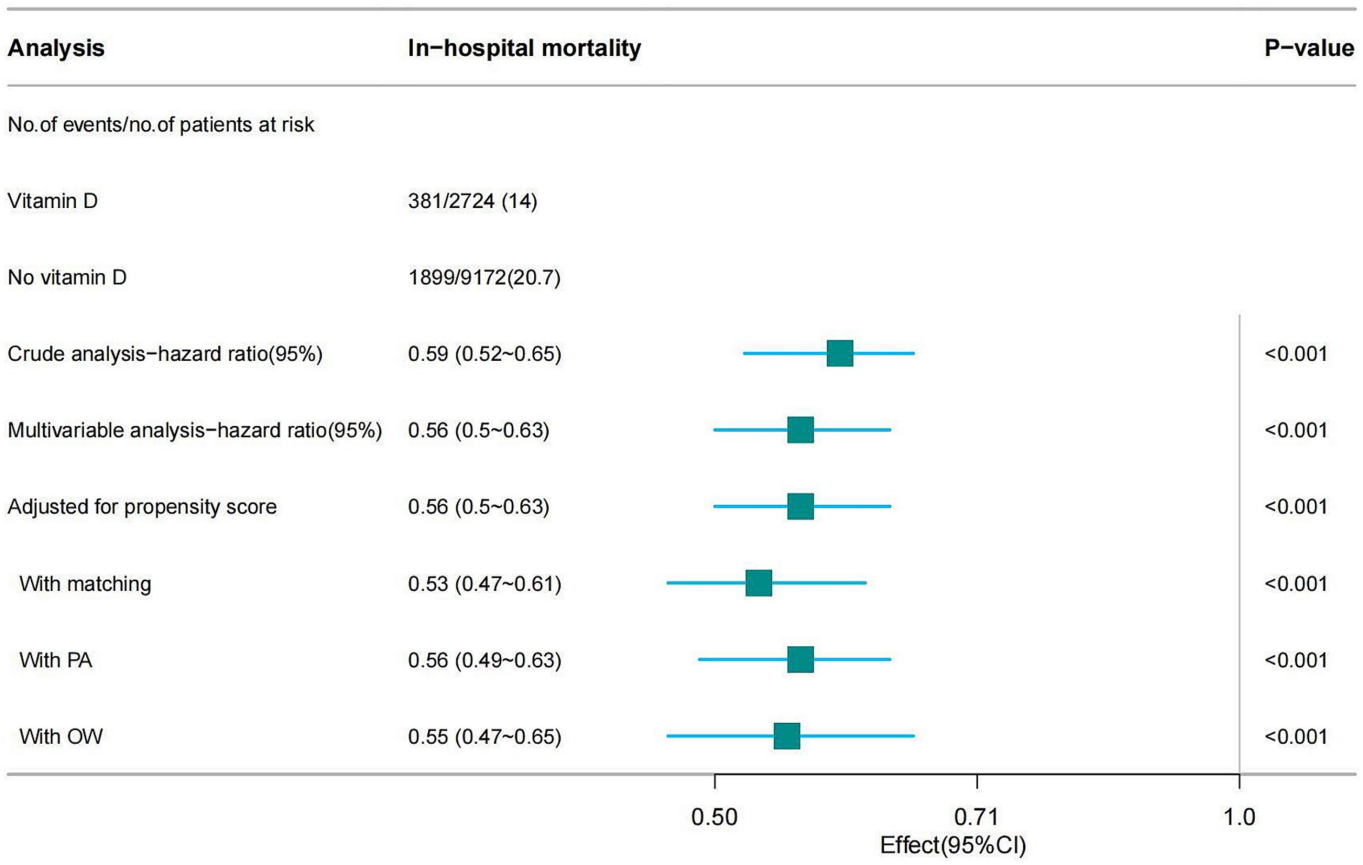
Forest plot shows HRs of in-hospital mortality in vitamin D group using a variety of models.

**Figure 3:**
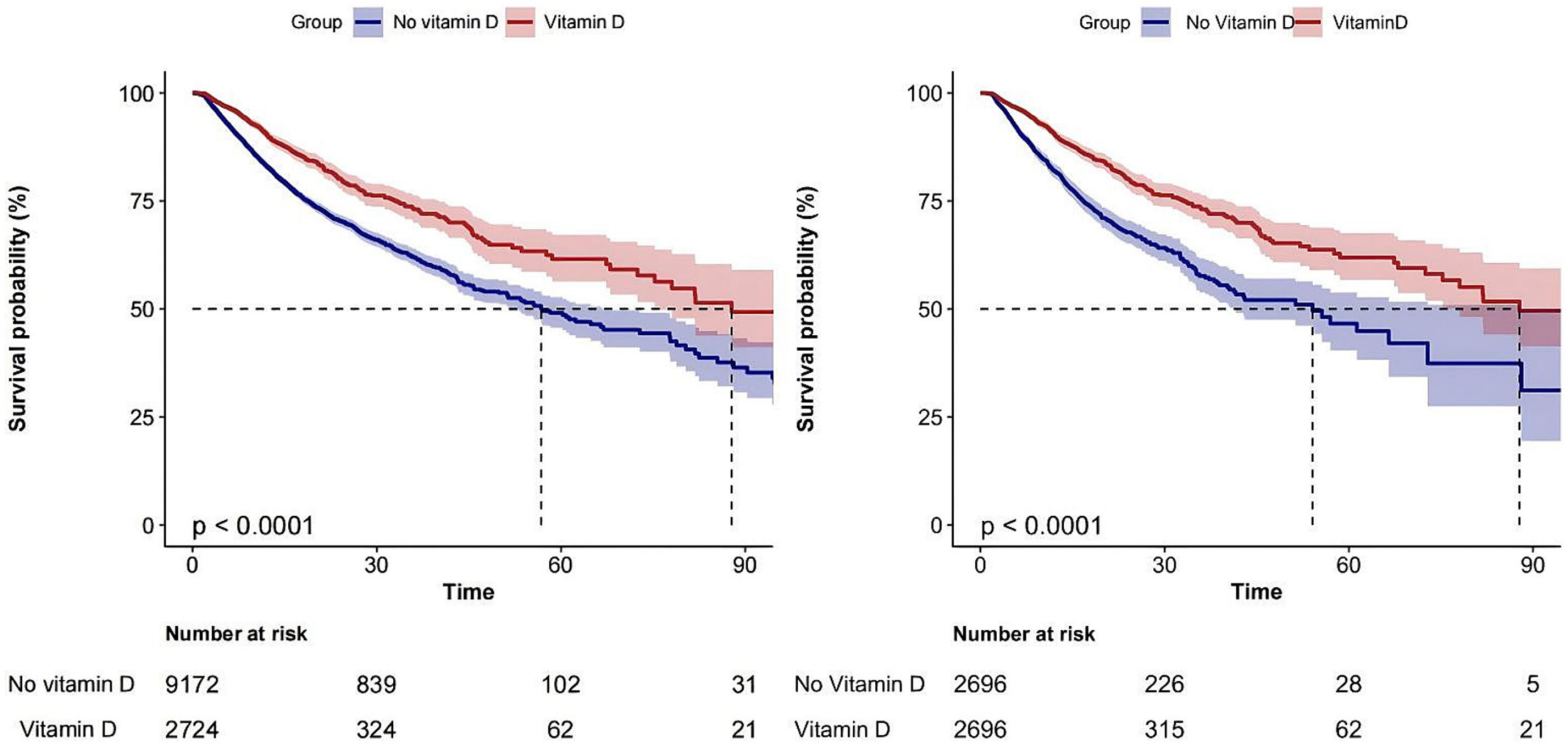
Kaplan–Meier survival curve for in-hospital mortality according to different groups.

The overall in-hospital mortality rate was 19.2%, and the in-hospital mortality rates for the vitamin D-supplemented group and the non-vitamin D-supplemented group were 14% (381/2724) and 20.7% (1899/9172), respectively (Figure 2) and the Kaplan–Meier curves showed that the vitamin D-supplemented group had a lower rate of in-hospital mortality (Figure 3).

In this study, univariate Cox regression analysis demonstrated that vitamin D supplementation significantly reduces the mortality risk in patients with SA-AKI, yielding a hazard ratio (HR) of 0.59 (95% CI: 0.52–0.65). This finding indicates that patients receiving vitamin D have an approximately 41% lower risk of death compared to those who do not receive supplementation. To account for potential confounding factors, we adjusted for all covariates listed in Table 1 during the multivariable Cox regression analysis, which resulted in a hazard ratio of 0.56 (95% CI: 0.50–0.63), suggesting the robustness of the results. Furthermore, we utilized PSM and adjusted for the propensity score, to mitigate the influence of confounders. The results revealed consistent hazard ratios of 0.56 (95% CI: 0.50–0.63) and 0.53 (95% CI: 0.47–0.61), reinforcing the assertion that vitamin D supplementation has a stable effect on reducing mortality risk. Lastly, the incorporation of the PA and OW methods further bolstered the reliability of our findings (see Figure 2). Notably, the E-value for this cohort ranged from 2.78 to 3.18, indicating a robust association between vitamin D supplementation and improved patient outcomes, thereby suggesting that this association remains significant even in the presence of potential unmeasured confounders.

### 3.4 Secondary outcome analysis with PSM cohorts

After adjusting for confounders using PSM and comparing the 28-day and 90-day mortality rates between the two groups, a statistically significant reduction in mortality was observed in patients who received vitamin D supplementation (16.5% vs. 26.3%, *p* < 0.001; 23.3% vs. 34.7%, *p* < 0.001). Furthermore, the vitamin D supplementation group exhibited a higher number of vasopressor-free days (21.7 ± 10.5 vs. 19.6 ± 11.9, p < 0.001) (Table 2).

**Table 2:** Secondary outcome analysis after matching.

Secondary outcomes	Total	Propensity-score–matched patients		*p*-value
		No vitamin D	Vitamin D	
28-day mortality, *n* (%)	1155/5390 (21.4)	710/2695 (26.3)	445/2695 (16.5)	< 0.001
90-day mortality, *n* (%)	1564/5394 (29.0)	936/2697 (34.7)	628/2697 (23.3)	< 0.001
Norepinephrine free day, Mean (SD)	5,394	19.6 ± 11.9	21.7 ± 10.5	< 0.001

### 3.5 Sensitivity analysis and subgroup analysis

We performed a sensitivity analysis on patients who developed AKI within 48 h of ICU admission. Utilizing univariate and multivariable Cox regression analyses, PSM, adjusted for propensity score, PA, and OW methods, we found that vitamin D supplementation significantly reduces mortality rates among these patients (Table 3).

**Table 3:** HRs of in-hospital mortality in vitamin D group using a variety of models.

Analysis	In-hospital mortality	*p*-value
No. of events/no. of patients at risk		
Vitamin D	350/2140 (14.1)	
No vitamin D	1728/8178 (21.1)	
Crude analysis-hazard ratio (95%)	0.57 (0.51 ~ 0.64)	<0.001
Multivariable analysis-hazard ratio (95%)	0.54 (0.47 ~ 0.6)	<0.001
Adjusted for propensity score	0.54 (0.48 ~ 0.61)	<0.001
With matching	0.54 (0.47 ~ 0.62)	<0.001
With PA	0.54 (0.47 ~ 0.62)	<0.001
With OW	0.53 (0.45 ~ 0.63)	<0.001

**Figure 4:**
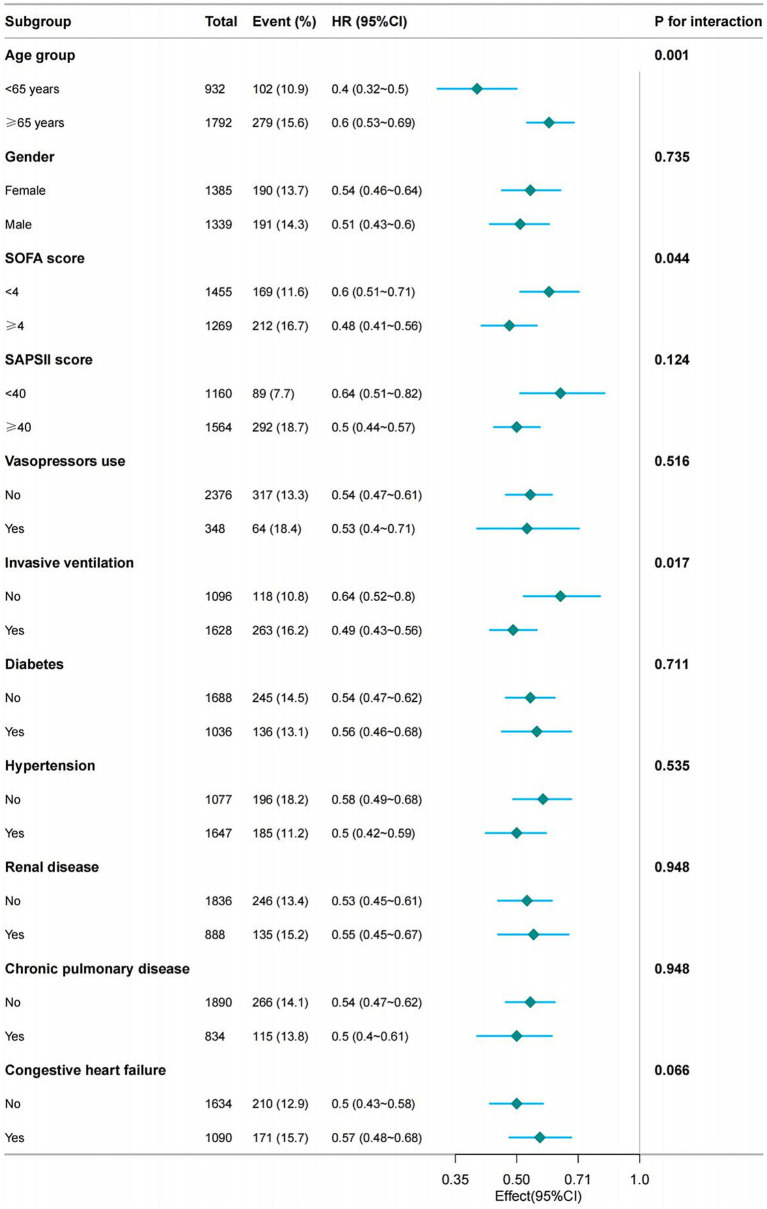
Forest plot shows HRs of in-hospital mortality in vitamin D group in subgroup analyses.

After adjusting for all covariates in Table 1, we conducted a subgroup analysis based on age, gender, SOFA score, SAPS II score, the use of vasopressor agents, the presence of invasive mechanical ventilation, and the existence of hypertension, diabetes, renal disease, chronic pulmonary disease, and congestive heart failure. The results remained stable across these subgroups. However, some interactions were observed concerning age, SOFA scores, and mechanical ventilation (Figure 4).

## 4 Discussion

Our study suggests that vitamin D supplementation during hospital admission is associated with a lower risk of in-hospital mortality in patients with SA-AKI. This association was further validated by PSM, PA, OW, sensitivity analysis and subgroup analyses. E-value analyses ranging from 2.78 to 3.18 indicate that unmeasured confounders are unlikely to negate the observed effects. The results confirm that vitamin D, as an inexpensive, readily available and relatively safe intervention, is associated with improved prognosis in SA-AKI, demonstrating the robustness and reliability of the findings. In addition, our findings suggest that vitamin D supplementation during hospitalization is not only associated with reduced in-hospital mortality, but also with reduced 28-day and 90-day mortality. At the same time, patients receiving vitamin D supplementation had a higher number of days without norepinephrine. These results provide further evidence to support the beneficial role of vitamin D in the treatment of SA-AKI.

The body uses two main forms of vitamin D: vitamin D_2_ (ergocalciferol) and vitamin D_3_ (cholecalciferol). These forms are obtained from the diet and synthesized in the skin. They are metabolized in the liver to form 25-hydroxyvitamin D. Subsequently, the enzyme 25-hydroxyvitamin D-1alpha-hydroxylase catalyzes its conversion in the kidneys to the active form, 1,25-dihydroxyvitamin D (6, 17). Although vitamin D has historically been associated primarily with skeletal metabolism, contemporary research is placing increasing emphasis on its effects on the non-skeletal system. Vitamin D deficiency has been linked to a wide range of diseases, including malignancies, immune system disorders (e.g., Crohn’s disease, rheumatoid arthritis), cardiovascular disease, depression and pulmonary dysfunction leading to asthma (15, 18–20). In addition, vitamin D deficiency has been linked to increased susceptibility to infection. In particular, vitamin D plays a role in modulating the immune response, restoring immune homeostasis and reducing organ dysfunction (21). Research indicates that vitamin D can inhibit cellular proliferation and promote the differentiation of various lineages, which is crucial for regenerating epithelial barriers and maturing immune cells. For example, lymphocytes, neutrophils, monocytes, and dendritic cells express the vitamin D receptor (VDR) and act as direct targets of 1,25(OH)₂D₃. Moreover, these cells facilitate the activation of circulating 25(OH)D₃ through the hydroxylation activity of the CYP27B1 enzyme (22). The immunoregulatory effects of 1,25(OH)₂D₃ are evident in its ability to switch between cell-mediated (Th1) and humoral (Th2) immune responses. Additionally, vitamin D enhances macrophage activation and stimulates the synthesis of antimicrobial peptides in epithelial and immune cells, which is vital for clearing bacterial and viral infections.

In critically ill patients, vitamin D serves a significant role as a natural vitamin. The immune response in sepsis functions as a double-edged sword: while an effective immune response is essential for combating infection, an excessive or dysregulated response can result in tissue damage and organ dysfunction. As such, the immunoregulatory function of vitamin D has emerged as a potential therapeutic target. Vitamin D helps maintain immune homeostasis during sepsis and enhances the body’s antibacterial capacity by modulating the functions of immune cells, including T lymphocytes, B lymphocytes, and macrophages, regulating cytokine production, and influencing innate immunity (23). For example, T. Greulich et al. elucidated its role in attenuating the inflammatory response and enhancing the antimicrobial activity of innate immune cells, potentially linking deficiency to increased susceptibility to systemic inflammatory response syndrome (SIRS) and sepsis (7). In addition, Prakash Vipul et al. found an inverse association between vitamin D levels and length of hospital stay in septic patients, suggesting an increased risk of mortality in the intensive care setting due to vitamin D deficiency (8). Similarly, research by Megan A et al. highlighted a significant increase in 30-day mortality in septic patients with vitamin D insufficiency (24).

In the context of sepsis, the kidney is particularly vulnerable, with AKI significantly increasing hospitalization rates and mortality risk (25). Vitamin D deficiency in septic patients contributes to AKI through mechanisms that go beyond immune dysfunction. Vitamin D depletion upregulates the renin-angiotensin-aldosterone system (RAAS) and increases the expression of renal angiorenalin mRNA, precipitating AKI (26). In addition, vitamin D insufficiency exacerbates ischemia/reperfusion injury by impairing renal vascular function and accelerates the progression of AKI to chronic kidney injury through modulation of the transforming growth factor-beta-1 signaling pathway, along with reduced expression of the vitamin D receptor (VDR) and Klotho protein (27, 28). Studies by David E. Leaf show a significant inverse association between bioavailable 25(OH)D levels and mortality in AKI patients, adjusting for age and blood creatinine (29). Lingyun Lai’s research also shows that 1,25-dihydroxyvitamin D levels decrease with increasing severity of AKI. In addition, low vitamin D levels are identified as a risk factor for AKI and are associated with a poorer prognosis once AKI manifests (30). Lynda K. Cameron et al. suggest that critically ill patients with moderate to severe AKI have significantly lower serum 1,25(OH)_2_D concentrations than those without AKI, and that recovery from AKI correlates with increased serum 1,25(OH)_2_D levels. Early assessment of vitamin D status and supplementation may therefore attenuate the progression of kidney disease and improve patient outcomes (31).

Despite numerous studies investigating the utility of vitamin D as a therapeutic intervention for SA-AKI, there is ongoing debate about its efficacy in improving patient outcomes. Evidence from studies investigating vitamin D supplementation during hospitalization has demonstrated a significant reduction in in-hospital mortality in patients with chronic obstructive pulmonary disease (COPD), suggesting potential benefits that extend to patients with sepsis (32, 33). For example, Tzu-Hsien Liao et al. have proposed that appropriate vitamin D supplementation may attenuate the progression and severity of AKI in animal models, although translation to humans remains to be validated (34). Preclinical studies have also highlighted the immunomodulatory effects of vitamin D in attenuating lipopolysaccharide-induced oxidative stress and renal expression of inflammatory cytokines, particularly relevant in SA-AKI (28, 35). The present study uses data from the extensive MIMIC database to analyze the association between vitamin D supplementation on admission and prognosis in critically ill SA-AKI patients, showing a reduced risk of mortality with early vitamin D administration. Given the high prevalence of vitamin D deficiency in critically ill populations, particularly those with underlying chronic kidney disease, adjustments for confounding variables were carefully applied in subgroup analyses to ensure robust results. These findings highlight the potential of timely and appropriate vitamin D supplementation not only to modulate immune responses and enhance antimicrobial defenses, but also to attenuate the progression of ischemia-reperfusion kidney injury and improve patient prognosis.

This study is the first to examine the relationship between vitamin D supplementation and prognosis in SA-AKI patients admitted to intensive care. Rigorous statistical methods were applied to ensure robust results, taking advantage of the large sample size from the MIMIC-IV database. However, several limitations should be noted. The retrospective design precluded access to baseline and post-treatment vitamin D levels, which may have limited the findings. Despite efforts to control for confounding variables, residual confounding may still exist. In addition, the study focused exclusively on the binary presence of vitamin D supplementation, without exploring optimal dosing or administration protocols. Future prospective studies are needed to clarify the most effective strategies for vitamin D supplementation in this patient population.

## 5 Conclusion

Vitamin D supplementation has been demonstrated to reduce in-hospital mortality, as well as 28-day and 90-day mortality, in patients with SA-AKI in the ICU, while also increasing the number of days without norepinephrine administration within the 28-day period. This cost-effective and safe intervention involves testing vitamin D levels in critically ill patients and initiating supplementation promptly, potentially improving patient outcomes. However, further clinical trials are needed to provide definitive evidence of its efficacy in improving the prognosis of SA-AKI patients hospitalized in ICUs.

## Data Availability

The raw data supporting the conclusions of this article will be made available by the authors, without undue reservation.

## Footnotes

^1^http://www.example.com, R Foundation.
